# Investigating geographical accessibility and site suitability of medical laboratories in Kermanshah-Iran

**DOI:** 10.3389/fpubh.2022.1004377

**Published:** 2022-12-16

**Authors:** Ali Almasi, Sohyla Reshadat, Alireza Zangeneh, Mehdi Khezeli, Nader Rajabi Gilan, Shahram Saeidi

**Affiliations:** ^1^Public Health School, Social Development and Health Promotion Research Center, Health Institute, Kermanshah University of Medical Sciences, Kermanshah, Iran; ^2^Social Development and Health Promotion Research Center, Health Institute, Kermanshah University of Medical Sciences, Kermanshah, Iran

**Keywords:** accessibility, health services, spatial analyses, geographic information system (GIS), medical laboratory centers (MLCs)

## Abstract

**Introduction:**

One of the major challenges in developing countries is the inappropriate spatial distribution of medical laboratory centers (MLCs) which can lead to injustice in access to health services. This study aimed to investigate the accessibility to and site suitability of MLCs in Kermanshah Metropolis by GIS.

**Materials and methods:**

In this cross-sectional study, data were collected from the Iran Statistical Center and Deputy of Treatment of Kermanshah University of Medical Sciences. We used Arc/GIS 10.6 software, AHP technique, and network analysis tools to determine the access status of Kermanshah citizens to MLCs in 2019 and site selection for MLCs. The layers used in this study included population density, city development trends, compatible and incompatible land uses, pathways, land slope, river area, and access radius.

**Results:**

About 70% of households had inappropriate access to all MLCs in walking scenario. This ratio was 31.26% for 5 min, 9.58% for 10 min, and 6.09% for 15 min driving. Comparisons between public and private MLCs showed that in walking scenario, 88% of households had improper access to public and 80% to private MLCs. Based on 5 and 10 min of driving, 57 and 19% of households had inappropriate access to public MLCs, and 45 and 17% to private MLCs, respectively. Also, with 15 min of driving, 8% of households had improper access to public and 18% to private MLCs. Findings showed that scores provided for population density criteria were (0.298), distance from existing laboratories (0.213), proximity to pathways (0.175), consistent land use (0.129), city development trend (0.087), distance from riverfront (0.053), distance from incompatible land uses (0.015), and land slope (0.03). The final model was obtained by overlaying the layers. The model showed a 9-degree range from very bad to very good in Kermanshah city for the construction of laboratory centers (CR<0.01).

**Conclusion:**

The site selection model showed that the location of the proposed centers can be in the north and outskirts of the city to facilitate citizens' access to the MLCs. These results emphasize the justice in the spatial distribution of MLCs for the benefit of deprived populations as a global value.

## Introduction

Inappropriate spatial distribution of healthcare facilities (HCFs) is still common throughout Iran (1, 2), while balanced access of communities to health services is especially important to ensure social justice and equality (3). The increase in population, the growth of chronic diseases, and the improvement of the quality of life in recent years have increased the demand for the construction of new health service centers. Meanwhile, the minimum requirements for establishing a new health service center, including budget and optimal location, must be met (2, 4). Inequality in access to HCFs due to inappropriate spatial distribution in a particular country or geographic region is one of the weaknesses of governance and can lead to widespread public dissatisfaction (5, 6). Therefore, optimizing the spatial distribution of health services according to urban developments, and people's access to the nearest service center in the shortest possible time can be effective in public satisfaction (7, 8).

There is growing evidence, that with the use of geographic information system (GIS), and various statistical approaches, the detection of Spatial distribution of diseases, and susceptible areas (optimal location) have been identified in various fields such as medical geography, natural geography (9–14), and access to health centers better than before. GIS is now a valuable tool in ensuring access to vulnerable and disadvantaged populations to health care services. It is also used to the planning, monitoring, and evaluating the health systems as a useful technology for collection, storage, processing, analysis, and visualization of geospatial data, especially in developing countries (15, 16). In 1999, the UK Department of Health and Human Services conducted a study on the strategic development of health care centers regarding the location of health services (GIS) (17), and then through studies on health geography and GIS applications, the role of location analysis for constructing health services has become more prominent (18).

It seems that there are few studies on geographical access to medical diagnostic centers (laboratories) and providing a model for the construction of new centers, especially in Iran (19, 20). The laboratories in Iran are privately or publicly owned. This situation is different from the distribution of laboratories in developing countries, which indicate the need for conducting this study. On the other hand, Kermanshah metropolis located in the west of Iran is one of the semi-developed cities in terms of access to health services and has faced with inequality in access to health services (4, 18, 21, 22). Also, this study can be effective in assessing the accessibility to and providing a model for the construction of new laboratory centers, and in the future studies, and be a guidance and reference point for researchers in future studies.

### Literature review

The studies show that healthcare centers and facilities are inequitably distributed (5, 15, 16, 23). Health care site selection is one of the ill-structured problems which is faced by planners, especially in developing countries like Iran (2, 4, 24–27). Some studies also investigate the site selection and locating of Healthcare facilities using a range of software and criteria. Askari et al. indicated that only about half of Yazd city (Iran) residents have proper access to hospitals and the distribution of hospitals and beds in different regions of Yazd was inequitable. They also concluded that the numbers and space of hospitals in Yazd were sufficient, however, hospitals are inaccessible to a significant portion of the urban population (1). A study by Reshadat et al. showed residents had not equal access to hospitals, and also their distribution was not proportionate with population distribution and households. There is inequality in access to medical facilities focused on hospitals in Kermanshah (Iran) (28). Lao et al. concluded that the spatial accessibility index values for the community of Tacloban City in the Philippines were generally low. A new suggested site resulted in better interaction probability, a higher supply-demand ratio, and higher spatial accessibility indices (15). A study by Parvin et al. showed revealed that spatial discrepancy exists in the study area in terms of access to healthcare facilities and achieving equal healthcare access in Murshidabad, India (16). Proposed the GIS-based approach for Healthcare centers site selection. However, there are a few studies conducted the Laboratory center sites (29). A study in Ghana investigated the distribution of primary healthcare clinics and found that only 15% of those had proper access to the nearest referral health facility. They also concluded that the PHC clinics were spatially distributed at random rather than clustered, and the hospitals or medical laboratories were spatially dispersed (29). Site selection supported by GIS could have an essential function in creating an environment to solve spatial data problems (5). Though GIS and MCDA are two diverse areas of study, their incorporation could profit the complexity of location selection by investigating the geographical decision and assessing the order of other factors (30, 31). AHP is among the most-used MCDA methods for site selection (23, 24).

The results of the study by Almansi et al. showed The proposed model was applicable and appropriate for assessing the suitability of a hospital site and revealed that the MLP model is reliable and consistent with the AHP. It is a sufficiently promising approach to the location suitability of hospitals to ensure effective planning and performance of healthcare delivery in Malacca, Malaysia (5). Reshadat et al. showed the spatial distribution of health centers (in terms of the radius of access) and compatibility of the land-use were not properly considered over the 15-year period. They also concluded that by using GIS and AHP to provide health coverage for the current population in the city of Kermanshah, 13 new health centers are needed in suitable locations (2). Site selection studies, such as Hospital Site Suitability in Iran (24), and healthcare site selection in West Bengal (6) have validated that MCDA could be the most suitable model for solving site-based issues.

## Materials and methods

### Study area

Kermanshah is one of the western provinces of Iran and comprises 14 cities. Kermanshah Metropolis is the capital of Kermanshah Province. According to the 2016 census, the Kermanshah metropolis has a population of 937,527 and an area of over 10,000 hectares. In recent years, the Kermanshah metropolis has been facing several issues such as inadequate access elderly, a population of 15–65 years, Children to Health Care Centers, Clinical Laboratories, Hospitals, and Emergency Centers (2, 32, 33).

### Data description and GIS techniques

#### MLCs sites

In this study, we prepared statistical blocks and MLCs information statistical blocks are provided as shape-file in ARC/GIS software by Iran Statistics Center and contain population information based on the latest census (2016) in Iran. Information about the laboratories, including the number, type of dependence (private or governmental), and the laboratory locations provided by the Kermanshah University of Medical Sciences. In this study, all of the diagnostic laboratories in the private sector or public and private hospitals and clinics were included in the study. Kermanshah metropolis had 50 active laboratories, of which 21 were governmental and 29 were private laboratories. In the following, the laboratories based on the address were located on statistical blocks by Arc/GIS10.6. Then the layer of passages was digitized in Arc/Map environment, and topology and spatial relationship between passages were created in Arc/Catalog environment., using the Network Analyze tool in the Arc/Map environment, the access time was calculated by walking and vehicles separately (using public or private transportation). The distance from the home of the people to the nearest laboratory was calculated both by walking and by using public or private transportation. In technical calculations, the walking speed of a person in normal mode is considered to be between 0.75 and 1.25 meters per second (m/s) (20). Also, the radius of the standard access to laboratory facilities in Iran has been set at 700 meters (21). Therefore, in the present study, to calculate the access time by walking, the speed of a person was considered to be 1 meter per second (i.e., each person can walk 60 meters in 1 min), and according to the defined standard radius and the speed ratio of each person, the time of 11.66 min walking from home to the lab was used in the calculations as the basic access time (Equations 1 and 2). It is noteworthy that in this study, adequate access means that people can access the laboratories by walking in 11.66 min, and inappropriate access means that people live in areas that cannot access the laboratory within 11.66 min of walking.

Equation1:

The speed of each person is one meter per second: V = 1m / sec

The speed of each person in one minute is equal to 60 meters: V = 60 m / min

Equation2:

How to calculate the walking time


$$\begin{array}{l} t=\frac{s}{v}\;\frac{700\;m}{60\;m}=11.66\;minutes \end{array}$$

In which T = time, S = distance, V = speed per person per minute.

To calculate the appropriate and inappropriate access with vehicles, a Geo-database was first established in Arc/GIS. Then, the road layer was divided into three groups: first-class roads (highways), second-class roads (inner-city roads), and local streets (including streets and alleys at the neighborhood level). This division was determined based on the standards defined in Iran and the rules of car speed in all three types of roads, according to which the speed of cars on highways was 60 kilometer, and on second-class roads and local streets, 30 kilometer (20). Then, using the Network Analyze tool, the areas covered by the MLCs were determined based on driving within 5, 10, and 15 min; as a result, the areas with adequate access were identified and the areas outside these determined areas were defined as inappropriate access (15, 21, 22). In the following, areas with inappropriate access were identified in Arc / GIS10.6 software using the Erase tool. Also, using the Clip tool, the areas that had adequate access were identified by private and governmental laboratory centers. For this purpose, polygons that were formed as boundaries around the laboratories were identified as Features Erase and Clip Features, and statistical blocks were considered as the main layer on which the cuts were made.

#### Site selection process

Using the previous studies (20–22) and the opinions of experts and specialists in the field of health policy and management, and urban planning (a total of 15 experts), the effective layers in locating laboratories were determined. These layers included: A- Population density (this indicator is one of the important indicators for the construction of laboratory centers. This means that in places with higher population density, more laboratories should be built compared to the other areas) (27), B- City development process, C- pathways, D- Land slope (areas with a slope of 0.5–6% were considered as desirable areas for construction of laboratory centers) (28), E- River area, F-radius Access (distance from existing laboratories, which in this study was considered a radius of 750 meters), and G- Compatible uses and incompatible uses (uses that are in the sphere of influence of each other, must be consistent in terms of compatibility and activity with each other, and do not interfere with other activities. For example, uses such as Health centers were classified as compatible uses, residential centers were classified as neutral, and educational and industrial centers were classified as incompatible (27). Criteria classification in terms of distance was done by the Euclidean Distance tool in the GIS environment. The created distance maps were raster maps, in which the distance of each pixel in the map indicated the distance as a straight line to the places under study. Pairwise comparisons between the main criteria in locating laboratories were performed using the opinions of experts and previous studies. As a result, experts compared the criteria and determined the score of each criterion relative to each other. These comparisons were based on a 9-quantity Tomas L Satty chart in which weighed 1 as (equally preferred), 3 (moderately preferred), 5 (strongly preferred) 7 (very strongly preferred), 9 (extremely preferred), and weights of 2, 4, 6, and 8 were considered as intermediate values. In this study using the hierarchical analysis process (AHP). The AHP technique is one of the most comprehensive systems designed for multi-criteria decision making, as it enables the formulation of issues hierarchically. The basis of the AHP technique is based on pairwise comparisons and determining the degree of priority of the elements over each other according to the desired criteria and is used to solve multi-criteria evaluation problems and determine the priority of several options according to the desired criteria. Inconsistency rate “CR” is an indicator that measures the consistency of experts' responses to pairwise evaluations and comparisons. The inconsistency rate also shows how much confidence can be given to the priorities of the comparisons. According to Saaty, if the inconsistency ratio is lower than 0.1, the consistency of comparison matrices is approved and acceptable, but if the inconsistency rate is >0.1, it indicates a discrepancy in the assessments and judgments of experts (24). The distance maps changed to weighted (fuzzy) maps. We recruited Fuzzy after AHP, using the spatial analysis tools in GIS. In the last step, the Ext_AHP extension was used in Arc/Map software to meet the final location model. The Ext_AHP extension calculates the value of each of the criteria according to the pairwise comparison matrix formed in the GIS, and by overlapping the score of the layers with the other layers, the final model in the form of Raster maps was extracted in Arc / GIS environment to locating laboratory centers.

## Results

**Figure 1:**
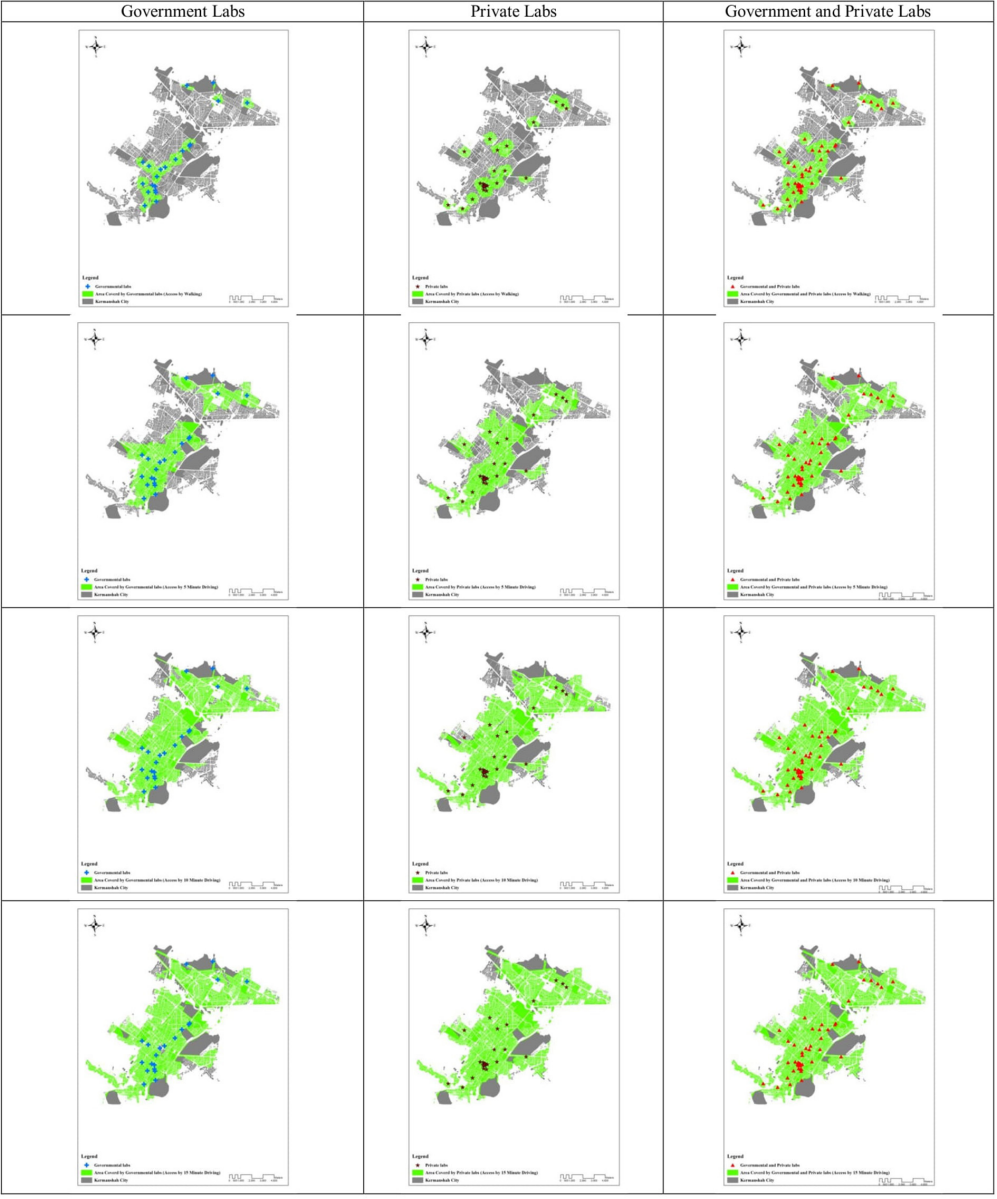
Area covered by Government al and private Labs.

Findings showed that about 70% of households had inappropriate access to laboratories (governmental and private) within 11.66 min. This ratio was 31.26% for 5 min driving (using public or private transportation), 9.58% for 10 min driving, and 6.09% for 15 min driving. Comparisons between governmental and private laboratories showed that within 11.66 min walking, 88% of households had inappropriate access to governmental and 80% to private laboratories. Based on 5 and 10 min driving, 57 and 19% of households, respectively, had inappropriate access to governmental laboratories, and 45 and 17% to private laboratories, respectively. Also, with 15 min of driving, 8% of households had inappropriate access to governmental and 18% to private laboratories (Figure 1 and Table 1).

**Table 1:** Comparison of appropriate and inappropriate access to Governmental and private Labs in Kermanshah.

**Laboratory Centers**			**Households with appropriate and inappropriate access by walking**		**Households with appropriate and inappropriate access by 5 min driving**		**Households with appropriate and inappropriate access by 10 min driving**		**Households with appropriate and inappropriate access by 15 min driving**	
			**Households**	**Population**	**Households**	**Population**	**Households**	**Population**	**Households**	**Population**
Governmental Labs	Appropriate access	*N*	33509	109578	126500	407041	232933	760000	260789	856428
		%	11/70	11/57	44/15	42/99	81/31	80/28	91/03	90/47
	Inappropriate access	*N*	252975	837073	159984	539610	53551	186651	25695	90223
		%	88/30	88/42	55/84	57/01	18/69	19/72	8/97	9/53
	Total	*N*	286484	946651	286484	946651	286484	946651	286484	946651
		%	100	100	100	100	100	100	100	100
Private labs	Appropriate access	*N*	56744	183042	157296	508027	235727	771180	234382	773040
		%	19/80	19/33	54/90	53/66	82/28	81/46	81/81	81/66
	Inappropriate access	*N*	229740	769609	129188	438624	50757	175471	52102	173611
		%	80/19	80/66	45/10	46/34	17/72	18/54	18/19	18/34
	Total	*N*	286484	946651	286484	946651	286484	946651	286484	946651
		%	100	100	100	100	100	100	100	100
Government al and private Labs	Appropriate access	*N*	84352	270092	196935	640047	259035	849475	269048	885741
		%	29/44	28/53	68/74	67/61	90/42	89/74	93/91	93/56
	Inappropriate access	*N*	202132	676559	89549	306604	27449	97176	17436	60910
		%	70/56	71/46	31/26	32/39	9/58	10/26	6/09	6/44
	Total	*N*	286484	946651	286484	946651	286484	946651	286484	946651
		%	100	100	100	100	100	100	100	100

Findings obtained from expert opinions and pairwise comparisons between layers showed that scores provided for population density criteria were (0.298), distance from existing laboratories (0.213), proximity to pathways (0.175), consistent land use (0.129), city development trend (0.087), distance from riverfront (0.053), distance from incompatible land uses (0.015), and land slope (0.03). Accordingly, a CR value < 0.01 was significant and consistency between judgments was confirmed.

**Figure 2:**
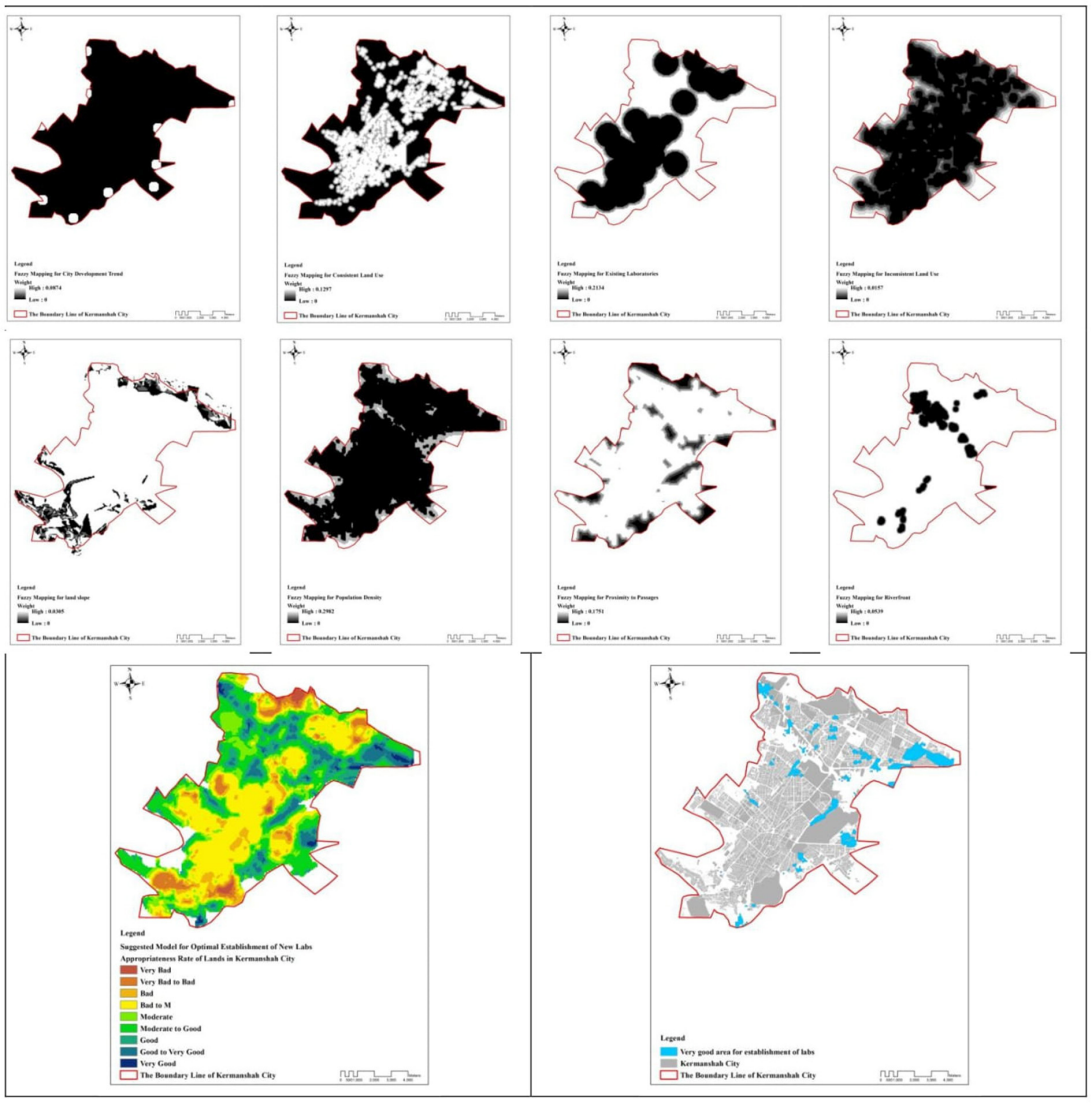
Fuzzy mapping for layers and suggested model for optimal establishment of new labs.

Figure 2 presents the fuzzy and weighted 8-step criteria. In these maps, the highest weight was indicated in white, and the black was the lowest weight. After evaluating and then combining the layers with the Ext_AHP extension in ArcGIS software, the model for improving access was obtained. Accordingly, the lands of Kermanshah were determined in a 9-degree range (very bad, very bad to bad, bad, bad to medium, medium, medium to good, good, good to very good, very good) to improve the access of citizens and build new MLCs in the future. Eventually, the lands that were very good for the construction of MLCs were marked with blue, most of which were located at the north of Kermanshah metropolis. Another important point in map 2 is that the suburbs have a larger share of the proposed locations.

## Discussion

This study aimed to determine the status of access to MLCs and provide a model for locating MLCs using GIS in Kermanshah metropolis in the west of Iran. The results showed that more than two-thirds of households had inappropriate access to MLCs by walking. However, the population with inappropriate access to the MLCs during a 15-min driving using public or private transportation was 10%. It seems that the problem of poor access through walking to different levels of healthcare services is a general challenge in the world. A study in Rwanda also showed the lowest level of accessibility in walking scenario, where only 26% of people had acceptable access to health facilities (34). A study in Yazd, Iran showed that only about half of the people of Yazd have proper access to different hospital wards (1). The results of a study showed that the percentage of the population without geographical access to Kermanshah hospitals (which all include lab) in walking scenario was 68.80, 64.23, and 66.20%, respectively, in 1997, 2007 and 2012, respectively (22). Another study in Iran by Reshadat et al. also demonstrated that only 37% of the individuals under 19 years had appropriate access to hospitals and emergency centers (33). Although in the present study, the results indicated that access by vehicles is better than walking, but it should be noted that even with vehicles, 16% of Kermanshah population do not have adequate access to MLCs. Since there are 50 active laboratories in the city of Kermanshah, it seems that the lack of proper geographic distribution of these centers has caused some people to not have access to these centers, even by spending 10–15 min using vehicles.

The results showed that private MLCs covered more households than public laboratories. The greater number of private centers and their proper geographic distribution (29 vs. 21) can justify better access to these centers. Public laboratories are mostly concentrated in public hospitals. This shows that public hospitals do not have a proper geographic distribution in Kermanshah. At the same time, the proximity of diagnostic services with medical services is the best state of providing healthcare, which should be provided through hospitals, especially public ones. In urban development plans, diagnostic services differ from healthcare services and are evaluated in the context of commercial services. In other words, out-of-hospital laboratories comply with the regulations of commercial centers, and in contrast, laboratory of clinics affiliated to the Ministry of Health and hospitals comply with the governmental regulations. Naturally, due to the lower cost of government centers, people are more willing to use these centers, while the present study showed that access to these centers is less. Therefore, to ensure proper access of people to laboratories, it is necessary to evaluate the geographical distribution and location. Locating MLCs requires a unified program for evaluating both public and private labs as well as sustainable urban development plan (35).

In this study, we proposed a model for MLCs in Kermanshah urban area. First, our results showed that in terms of site suitability, the lands in the urban area of Kermanshah can be evaluated in a 9-part spectrum from very bad to very good, something that was also concluded in previous studies (36, 37). In this study, the population density was the most important criterion for MLCs site selection, consistent with a similar study in Iran (38). In the current situation, the concentration of health services depends on the population density and vice versa. In other words, health services are sometimes established in densely populated areas to be available, and sometimes the population to enhance the accessibility settles close to health facilities (27). Meanwhile, people's health should be a priority regardless of how much the population is concentrated in one area. It is necessary to know that the city of Kermanshah has faced the phenomenon of marginalization in the last three decades due to the massive migration of people from the surrounding counties, and it has a high rank of informal settlement at the national level (2, 39). Therefore, it seems that the results of this study regarding the site selection for MLCs in the marginal areas of Kermanshah city can be justified.

In the site selection model of the current study, the presupposition was the lack of close proximity of the centers and ensuring their greater dispersion. This makes it impossible to build laboratories in the privacy area and without health-treatment (40), therefore, it provides fair access to medical diagnostic laboratories for all residents of the city.

In this study we used AHP method to site selection. Another model used to determine the appropriate location in other studies (41, 42) is the ANP method. Both AHP and ANP models are used to prioritize elements and are based on pairwise comparisons. Because we only used the criteria but not options (for example, the cities of a province as options) we could not use the ANP model. We used AHP because it is simple and understandable and is easily accessible in the GIS (AHP20 extension). Also it has a well-defined structure, and the regular target sequence, criteria, and sub-criteria. The model proposed in the present study can be considered as a general model for geographical access of the population to MLCs in all cities of Iran and other developing countries. This proposed model can be changed according to the contextual realities, including environmental and local assessments because some legal and economic factors can affect the applicability of the proposed locations.

One of the methods that could be used in this study to analyze the data is the E2SFCA method, which has been emphasized in similar studies such as McGrail and Humphreys (43), Luo and Qi (44), and Ngui and Apparicio (45). This was one of the limitations of our study. In future studies, the ability of this method in data collection and data analysis can be considered. There were some limitations to this study including lack of control over the time of access to the laboratories which was influenced by factors such as daylight hours and weather, failure to determine the impact of traffic volume and number of traffic lights, and not distinguishing between population with private vehicle and those used the public transportation. Also, this study did not consider economic conditions such as income that could affect the access rate.

Despite the mentioned limitations, this study had notable strengths. In the present study, considering the results of the hierarchical analysis in weighting to effective layers, the importance of each layer is well-incorporated so that the disturbing factor has the least weight. On the other hand, the AHP technique performed better than other weighting methods in this study because it was user-centered and used expert opinions. Therefore, the results of the weight allocated to different layers confirm the efficiency of this method in the present study.

## Conclusion

The results of the present study showed that public and private MLCs are not accessible by walking for more than two-thirds of the population of Kermanshah metropolis. However, access to those was better by using vehicles. Therefore, it is necessary to fair distribute the centers to ensure proper access for all citizens. The site selection model showed that the location of the proposed centers can be in the north and outskirts of the city to facilitate citizens' access to the MLCs. These results emphasize the justice in the spatial distribution of MLCs for the benefit of deprived populations as a global value.

## Data availability statement

The original contributions presented in the study are included in the article/supplementary material, further inquiries can be directed to the corresponding author.

## Author contributions

AA, SR, and ShS: apprehended the idea. AZ designed and analyzed it. ShS, AA, MK, and NR: interpreted the results and drafted the manuscript. All the authors take responsibility for the integrity of the work as a whole from inception to the published article. ShS is the guarantor. All the authors read and approved the final manuscript.

## References

[1] Askari R Shafiee M Charrahi Z Almodarresi SA Afrazandeh SM. Investigating the level of access to hospital medical facilities using the geographical information system (GIS) in Yazd, Iran, in 2019. J Commun Health Res. (2020) 9:241–55. 10.18502/jchr.v9i4.4977

[2] Reshadat S Saeidi S Zangeneh A. Using a geographic information system to identify the number and location of new health centres needed in the city of Kermanshah, Islamic Republic of Iran. Eastern Mediter Health J. (2020) 26:888–98. 10.26719/emhj.20.022 32896883

[3] Carey G Crammond B Malbon E. Personalisation schemes in social care and inequality: review of the evidence and early theorising. Int J Equity Health. (2019) 18:170. 10.1186/s12939-019-1075-2 31694649 PMC6836323

[4] Almasi A Saeidi S Zangeneh A Khezeli M Salimi Y Soofi M. Geographical access of elderly to health care centers during a 20 years period (1996–2016): a Case Study of Kermanshah, Iran. J General Int Med. (2020) 29:rs.3.rs-1211792. 10.21203/rs.3.rs-1211792/v1 33078293 PMC8481429

[5] Almansi KY Shariff ARM Kalantar B Abdullah AF Ismail SNS Ueda N. Performance evaluation of hospital site suitability using multilayer perceptron (MLP) and Analytical Hierarchy Process (AHP) models in Malacca, Malaysia. Sustainability. (2022) 14:3731. 10.3390/su14073731

[6] Dutta B Das M Roy U Das S Rath S. Spatial analysis and modelling for primary healthcare site selection in Midnapore town, West Bengal. GeoJournal. (2021) 87:4807–36. 10.1007/s10708-021-10528-w 34720353 PMC8540883

[7] Simões PP Almeida RM. Geographic accessibility to obstetric care and maternal mortality in a large metropolitan area of Brazil. Int J Gynecol Obstetr. (2011) 112:25–9. 10.1016/j.ijgo.2010.07.031 21056416

[8] Beccaro M Costantini M Merlo DF Group IS. Inequity in the provision of and access to palliative care for cancer patients. Results from the Italian survey of the dying of cancer (ISDOC). BMC Public Health. (2007) 7:66. 10.1186/1471-2458-7-66 17466064 PMC1885253

[9] Ruidas D Pal SC Islam ARM Saha A. Characterization of groundwater potential zones in water-scarce hardrock regions using data driven model. Environ Earth Sci. (2021) 80:1–18. 10.1007/s12665-021-10116-8

[10] Ruidas D Chakrabortty R Islam ARM Saha A Pal SC. A novel hybrid of meta-optimization approach for flash flood-susceptibility assessment in a monsoon-dominated watershed, Eastern India. Environ Earth Sci. (2022) 81:1–22. 10.1007/s12665-022-10269-0

[11] Band SS Janizadeh S Chandra Pal S Saha A Chakrabortty R Melesse AM. Flash flood susceptibility modeling using new approaches of hybrid and ensemble tree-based machine learning algorithms. Remote Sens. (2020) 12:3568. 10.3390/rs12213568

[12] Pal SC Arabameri A Blaschke T Chowdhuri I Saha A Chakrabortty R. Ensemble of machine-learning methods for predicting gully erosion susceptibility. Remote Sens. (2020) 12:3675. 10.3390/rs12223675

[13] Chowdhuri I Pal SC Saha A Chakrabortty R Roy P. Evaluation of different DEMs for gully erosion susceptibility mapping using in-situ field measurement and validation. Ecol Informat. (2021) 65:101425. 10.1016/j.ecoinf.2021.101425

[14] Pal SC Chakrabortty R Arabameri A Santosh M Saha A Chowdhuri I. Chemical weathering and gully erosion causing land degradation in a complex river basin of Eastern India: an integrated field, analytical and artificial intelligence approach. Nat Hazards. (2022) 110:847–79. 10.1007/s11069-021-04971-8

[15] Lao MSR Paringit MCR Roleda SRL. GIS-based site suitability analysis for healthcare facility development in tacloban city, Philippines. Geomate J. (2022) 22:16–23. 10.21660/2022.92.162

[16] Parvin F Ali SA Hashmi S Khatoon A. Accessibility and site suitability for healthcare services using GIS-based hybrid decision-making approach: a study in Murshidabad, India. Spatial Informat Res. (2021) 29:1–18. 10.1007/s41324-020-00330-0

[17] Greengross P Grant K Collini E. The history and development of the UK National Health Service 1948 1999. Health System Resource Centre, United Kingdom (1999).

[18] Reshadat S Zangeneh A Saeidi S Teimouri R Yigitcanlar T. Measures of spatial accessibility to health centers: investigating urban and rural disparities in Kermanshah, Iran. J Public Health. (2019) 27:519–29. 10.1007/s10389-018-0966-9

[19] Sadighi J Hosseini A Mohammad K Mahdavi S Mirab SS Safadel N. Modeling geographical accessibility to medical laboratory services in Iran: methodology and its challenges. Payesh. (2015) 14:421–34.

[20] Mamdouhi AR Lavi M. The development a descriptive model of spatial access to public treatment services hinterland floating two-step method (Case Study: municipalities of region 10). Res Human Geogr J. (2013) 44.

[21] Rezaei S Ghazanfari S Kazemi Z Karyani AK. Access to healthcare facilities: case study of Kermanshah province. J Kermanshah Univ Med Sci. (2014) 18:416–25. 10.22110/jkums.v18i7.1854

[22] Reshadat S Zangeneh A Saeidi S Ghasemi S Rajabi-Gilan N Zakiei A. Inequalities in access to hospitals: a case study in the Islamic Republic of Iran 1997–2012. Eastern Mediter Health J. (2019) 25:119–26. 10.26719/emhj.18.061 30942476

[23] Tripathi AK Agrawal S Gupta RD. Comparison of GIS-based AHP and fuzzy AHP methods for hospital site selection: a case study for Prayagraj City, India. GeoJournal. (2021) 87:3507–28. 10.1007/s10708-021-10445-y 34075269 PMC8159725

[24] Galankashi MR Nasri E Helmi SA Arjmand MM. Hospital Selection Problem: An Integrated Analytic Hierarchy Process (AHP) and Fuzzy-TOPSIS Approach.

[25] Reshadat S Saedi S Zangeneh A Ghasemi S Gilan N Karbasi A. Spatial accessibility of the population to urban health centres in Kermanshah, Islamic Republic of Iran: a geographic information systems analysis. Eastern Mediter Health J. (2015) 21:389. 10.26719/2015.21.6.389 26369997

[26] Sadighi J Hosseini A Mohammad K Mahdav S Samiee SM Safade N. Geographical accessibility to medical laboratory services in Iran: Takab case study. Payesh. (2015) 14:647–65.

[27] Sadighi J Hosseini A Mohammad K Mahdavi S Mirab Samiee S Safadel N. Geographical accessibility to medical laboratory services in iran: the Qom case study. Payesh. (2016) 15:259–79.

[28] Reshadat S Saeidi S Sufi E Rjabi-Gilan N Ghasemi R. Investigating Inequalities in Access to Hospital Medical Facilities Using Geographical Information System in Kermanshah's Metropolitan Area. J Hospital. (2016) 15:9–22.

[29] Kuupiel D Adu KM Bawontuo V Mashamba-Thompson TP. Geographical accessibility to district hospitals/medical laboratories for comprehensive antenatal point-of-care diagnostic services in the Upper East Region, Ghana. EClinicalMedicine. (2019) 13:74–80. 10.1016/j.eclinm.2019.06.015 31517264 PMC6734000

[30] Tripathi AK Agrawal S Gupta R. A conceptual framework of public health SDI. In: Applications of Geomatics in Civil Engineering. India: Springer (2020). p. 479–87. 10.1007/978-981-13-7067-0_37

[31] Feizizadeh B Roodposhti MS Jankowski P Blaschke T. A GIS-based extended fuzzy multi-criteria evaluation for landslide susceptibility mapping. Comput Geosci. (2014) 73:208–21. 10.1016/j.cageo.2014.08.001 26089577 PMC4376179

[32] Almasi A Saeidi S Zangeneh A Khezeli M Salimi Y Soofi M. Geographical access of the elderly to health care centers during a 20-year period (1996–2016): a case study of Kermanshah, Iran. J General Int Med. (2021) 36:3249–51. 10.1007/s11606-020-06289-w 33078293 PMC8481429

[33] Reshadat S Saeidi S Zangeneh A Ziapour A Choobtashani M Saeidi F. The study of children and adolescents' access to hospitals and emergency centers in Kermanshah, West of Iran. Int J Pediatr. (2018) 6:7697–707. 10.22038/ijp.2018.29673.2609

[34] Huerta Munoz U Källestål C. Geographical accessibility and spatial coverage modeling of the primary health care network in the Western Province of Rwanda. Int J Health Geogr. (2012) 11:40. 10.1186/1476-072X-11-40 22984920 PMC3517388

[35] Tahari MMH Babaei MH Morovati SA. Investigation and ranking of Iranian provinces in terms of access to health sector indicators. Health Informat Manage. (2012) 9:356–69.

[36] Reshadat S Saedi S Zangeneh A Amooie MR Karbasi A. Equity in access to health care using geographic information system: a Kermanshah case study. J Mazandaran University Med Sci. (2014) 24:134–40.

[37] Skhiri H Bellaaj R Ben AN Ben HA. [Health indicators in Tunisia, trends in regional disparities over the last thirty years]. La Tunisie medicale. (2001) 79:92–7. 11414065

[38] Ebrahimzadeh I Ahadnezhad M Ebrahimzadeh AH Shafiei Y. Spatial organization planning of health services by the use of GIS: the case of zanjan city. Human Geogr Res Quart. (2010) 1:39–58.

[39] Habibi K Arefi M Doostvandi M Ashouri K. Reproduction of urban informality in Iran: its key factors, tools and challenges. J Urban Manage. (2022) 11:381–91. 10.1016/j.jum.2022.05.007

[40] Sadighi J Hosseini A Mohammad K Mahdavi S Mirab Samiee S Safadel N. Modeling geographical accessibility to medical laboratory services in Iran: methodology and its challenges. Payesh. (2015) 14:421–34.

[41] Mohammadi Zanjirani D Shahbandarzadeh H Ghorbanpour A Mohammadi Baghmollaie M. The application of fuzzy analytical network process (FANP) approach for appropriate location selection of health centers: case study in Ramsar cite. ISMJ. (2012) 15:127–36.

[42] Yaghfoori H Kashefi DD Ghader MJ. An analysis on dispersion and distribution of health centers and optimal locating of new clinics (Case Study: Piranshahr City). Environ Based Territor Plann. (2014) 7:129–48.

[43] McGrail MR Humphreys JS. Measuring spatial accessibility to primary care in rural areas: improving the effectiveness of the two-step floating catchment area method. Appl Geogr. (2009) 29:533–41. 10.1016/j.apgeog.2008.12.003

[44] Luo W Qi Y. An enhanced two-step floating catchment area (E2SFCA) method for measuring spatial accessibility to primary care physicians. Health Place. (2009) 15:1100–7. 10.1016/j.healthplace.2009.06.002 19576837

[45] Ngui AN Apparicio P. Optimizing the two-step floating catchment area method for measuring spatial accessibility to medical clinics in Montreal. BMC Health Services Res. (2011) 11:166. 10.1186/1472-6963-11-166 21745402 PMC3142205

